# Genetic contribution to mesenchymal stem cell dysfunction in systemic lupus erythematosus

**DOI:** 10.1186/s13287-018-0898-x

**Published:** 2018-05-24

**Authors:** Yantong Zhu, Xuebing Feng

**Affiliations:** Department of Rheumatology and Immunology, the Affiliated Drum Tower Hospital of Nanjing University Medical School, 321 Zhongshan Road, Nanjing, 210008 Jiangsu China

**Keywords:** Systemic lupus erythematosus, Mesenchymal stem cells, Genetic factors

## Abstract

Allogeneic mesenchymal stem cell (MSC) transplantation has recently become a promising therapy for patients with systemic lupus erythematosus (SLE). MSCs are a kind of multipotent stem cell than can efficiently modulate both innate and adaptive immune responses, yet those from SLE patients themselves fail to maintain the balance of immune cells, which is partly due to the abnormal genetic background. Clarifying genetic factors associated with MSC dysfunction may be helpful to delineate SLE pathogenesis and provide new therapeutic targets. In this review, the scientific evidence on the genetic contribution to MSC dysfunction in SLE is summarized.

## Background

Mesenchymal stem cells (MSCs), mainly located in the adult bone marrow, have a capacity for self-renewal and potential for multilineage differentiation into a variety of cell types, such as osteoblasts, chondrocytes, myocytes, and adipocytes [1]. These cells also provide a supportive microenvironment for hematopoietic stem cells, to maintain their growth, differentiation, and proper function [2]. Recently, MSCs have been demonstrated to display profound immunosuppressive effects on various immune cells [3] and thus are considered as a new therapeutic paradigm for autoimmune diseases.

Systemic lupus erythematosus (SLE) is a prototypic autoimmune disease characterized by the activation of both innate and adaptive immune responses. Although survival of SLE patients has improved considerably in the past decades, a substantial proportion of patients are still refractory to routine treatments and expected to have a poor prognosis. While allogeneic MSC transplantation results in the induction of clinical remission and improvement in organ dysfunction for patients with refractory SLE [4], evidence has shown that autologous MSCs are not beneficial for these patients [5], implying that abnormalities of MSCs themselves is involved in the progress of SLE disease.

As bone marrow (BM) MSCs separated from the in vivo environment of SLE still present impaired capacity to inhibit immune cells and induce peripheral tolerance [6], it is reasonable to mainly attribute MSC dysfunction to the intrinsic genetic defects in this disease. To further understand the regulatory mechanism of SLE MSCs, possible genetic factors implicated in the regulation of MSC function are discussed in combination with the latest research findings.

## Immunomodulatory effect of MSCs

Animal model studies and phase I/II clinical trials have demonstrated that MSCs have therapeutic potential in the treatment of a variety of autoimmune diseases, including SLE [7–10]. Although MSCs from different sources exhibited various immunomodulatory capacities in vitro [11], their efficacy is similar as reported in a clinical study using BM or umbilical cord-derived MSC transplantation for severe and refractory SLE patients [4]. However, the mechanisms by which MSCs affect immune cells and the underlying signaling pathways remain unclear. Recently, it has been proposed that MSCs may act through both paracrine secretion of soluble factors and cell–cell contact.

MSCs are associated with the inhibition of T-cell proliferation and upregulation of T regulatory (Treg) cells [12]. Evidence shows that murine MSCs secrete nitric oxide (NO) to inhibit T-cell production [13], and MSCs from mice with inducible nitric oxide synthase (iNOS) knocked down display impaired ability to prevent T-cell proliferation [14]. Besides, iNOS activation in MSCs has been shown to greatly inhibit the expansion of T follicular helper (Tfh) cells, a CD4^+^ T-cell subtype that helps B cells to generate affinity-matured antibodies, in lupus mice [15]. In human MSCs, the effects of NO is replaced by indoleamine 2,3-dioxygenase (IDO) [16], the production of which is enhanced by CD8^+^ T-cell-secreted interferon-γ (IFN-γ) [17], suggesting that different species may employ different effector molecules. Different from other T-cell subsets, CD4^+^CD25^+^FoxP3^+^ Treg cell levels are elevated after MSCs transplantation [5]. These cells could be induced from purified CD4^+^ T cells by allogeneic human MSCs in vitro, as mediated by prostaglandin E2 (PGE2) and transforming growth factor beta1 (TGF-β1) [18]. Through the upregulation of interleukin-10 (IL-10) and Fas ligand, CD8^+^ Treg cells could also be proliferated and functionally enhanced by MSCs [19]. Besides these soluble factors, cell–cell contact also plays an important role in MSC-induced T-cell regulation [15]. Recently, MSCs have been shown to be capable of transferring mitochondria into T cells [20], which may provide an explanation for how cell–cell contact regulates the immunomodulatory functions of MSCs.

MSCs participate in inhibiting B cell proliferation, differentiation, antibody production, and apoptosis [21]. It has been proven that MSCs stimulated with IFN-γ and tumor necrosis factor α (TNF-α) have enhanced regulatory effects on B cells [22]. Through programmed death 1(PD-1) and programmed death ligand-1 (PDL-1) interaction, IFN-γ may increase the amount of PDL-1 in MSCs to inhibit the proliferation and differentiation of B cells [23]. Meanwhile, MSC-derived chemokine C-C motif ligand 2 (CCL2) is also involved in B-cell proliferation, plasma cell differentiation, and immunoglobulin generation through the inactivation of signal transducer and activator of transcription 3 (STAT3) and induction of paired box protein 5 [24]. CCL2 was originally considered as a pro-inflammatory chemokine that helps recruit monocytes or macrophages to areas of inflammation. However, MSCs could turn this chemokine into an anti-inflammatory phenotype though matrix metalloproteinase (MMP)-induced proteolysis.

MSCs can reprogram macrophages and enhance their anti-inflammatory function [25, 26]. Macrophages are capable of being educated into alternatively activated macrophages (AAMs; also called M2), which possess enhanced phagocytic activity when co-cultured with MSCs, via up-regulation of IL-10/TGF-β1 and down-regulation of TNF-α/iNOS [27, 28]. These M2 macrophages express high levels of CD206 and have potent immunoregulatory function, characterized by enhanced inhibition of CD4^+^ T-cell differentiation and decreased ability to induce Th17 cell expansion. Recently, a role for IL-6 in MSC-driven macrophage polarization has been described in SLE patients [29].

Other immune cells, such as dendritic cells (DCs) and natural killer (NK) cells, are also regulated by MSCs. The maturation and antigen-presenting functions of DCs are suppressed by MSCs via soluble factors such as PGE2 and IL6 [30–32]. Meanwhile, IDO and PGE2 secreted by MSCs play a key role in MSC-induced inhibition of NK cells [33]. Interestingly, none of these soluble factors is solely responsible for the entire immune regulatory network of MSCs. Instead, a combination of molecules is usually required for MSC-mediated treatment effects.

## Impaired immune regulation of SLE MSCs

BM MSCs derived from SLE patients exhibit a lot of abnormalities compared with normal controls, including cytoskeleton-related defects [34] and increased cell senescence as well as apoptosis [35]. Their potential for differentiation and migration is greatly impaired [36]. Moreover, expression profiles of genes related to immune function in SLE MSCs, such as IDO, IL-6, IL-7, and TGF-β, are distinct from those in normal subjects [17, 37–39]. Correspondingly, the function of MSCs from SLE patients or lupus animal models is severely damaged and not sufficient to suppress various immune cells [40, 41], and may thus contribute to the onset of autoimmunity.

In SLE, due to the lack of efficient MSC regulation, the balance between Treg and Tfh cells is usually broken. As a consequence, peripheral Treg cells are decreased and Tfh cells are increased, especially in those with active disease [15, 38]. Evidence has shown that allogeneic MSCs could increase the number of peripheral Treg cells through the secretion of TGF-β in SLE patients [5, 42] and inhibit Tfh cell expansion via the activation of iNOS in lupus mice [15], indicating there is a restoration of Treg/Tfh balance after MSC treatment.

Similar to T cells, BM MSCs from SLE patients and lupus mice are less effective at preventing the proliferation and inhibition of B cells, as well as the secretion of autoantibodies, which is mainly mediated by reduced CCL2 production [6]. After the knock-down of the CCL2 gene, the ability of MSCs to suppress B cells is greatly impaired. Meanwhile, over-expression of CCL2 enhances the efficacy of MSCs to regulate B-cell proliferation, along with ameliorated renal pathology and reduced autoantibody production [6]. Besides CCL2, there is evidence showing that mice lacking PD-1 are capable of developing an SLE-like disease [43]. PD-1 reduction may hinder the cell-to-cell contact between MSCs and B cells and consequently inhibit B cell proliferation and differentiation [23].

Besides acquired immunity, the abnormality of innate immunity in SLE is also related to MSCs [40]. Macrophages from either lupus mice or SLE patients display decreased CD206 expression and inefficient phagocytic activity that could be corrected by MSCs from a healthy population in an IL-6-dependent manner [29]. Since IL-6 expression is reduced in BM MSCs from SLE patients [39], it is not surprising to find that SLE MSCs fail to regulate macrophage over-activation in vivo. Accordingly, impaired phagocytic activity of macrophages may result in the accumulation of apoptotic debris and lead to sequelae of autoimmune phenomena [44].

Recently the effect of MSCs on autophagy has been revealed. T cells from SLE patients appear to have higher levels of autophagy [20, 45], a basic cellular homeostatic process that enables cells to eliminate portions of their own cytoplasmic contents. Abnormal autophagic activity in SLE T cells is caused primarily by cellular accumulation of functionally defective mitochondria, which activates the autophagy process and subsequently promotes apoptosis of T cells [46]. Meanwhile, normal MSCs are capable of down-regulating autophagy in activated T cells through the inhibition of respiratory mitochondrial biogenesis [20].

## Genetic factors contributing to MSC dysfunction in SLE

Because MSCs from SLE patients still display impaired immunomodulatory function in vitro, it is assumed that the genetic background plays an essential role in MSC dysfunction. There is plenty of indirect evidence to imply genetic involvement in SLE MSC function (Table 1). Until recently, however, direct evidence of MSC genetic regulation in SLE has been reported in only a few studies.

**Table 1:** Factors that may be involved in the regulation of MSC function in SLE

Factor	Immunological effects	Reference
With direct evidence		
OAZ	Impair MSC regulation of B cells, leading to autoantibody production	[41]
Pbx1-d	Result in the generation of auto-reactive CD4^+^ T cells	[40]
p16^INK4A^	Inhibit TGF-β secretion, contribute to the reduction of Treg cells	[52]
With indirect evidence		
PD-1	Loss of peripheral self-tolerance in B cells	[23]
FcγRIIB	Acceleration of dendritic cell maturation	[55]
STAT1	Associated with IDO production	[60]
IFN γ	Stimulation of IDO production	[17]
IL-6	Macrophage polarization	[29]
IDO	Inhibition of T-cell proliferation	[17]
CCL2	Inhibition of B-cell proliferation and differentiation	[6]
PGE2	Inhibition of T-cell proliferation and monocyte differentiation	[30]
Galectin-3	Involved in T-cell proliferation	[64]
TNF-α	MSC migration	[61, 62]
Leptin	MSC senescence	[63]

### Genes directly resulting in MSC abnormality

Olfactory 1/early B cell factor-associated zinc-finger protein (OAZ) is a transcription factor involved in bone morphogenetic protein (BMP)-induced signaling pathways [47]. As a candidate susceptibility gene of SLE, it plays a role in regulating anti-nuclear antibody production in SLE patients [48]. OAZ is over-expressed in MSCs from SLE patients [41]. Knockdown of the OAZ gene with small interfering RNAs restores the ability of MSCs to suppress B-cell proliferation and terminal differentiation, followed by a decrease in anti-nuclear antibody production. The effect of OAZ is mediated by the chemokine CCL2, as the level of CCL2 was increased after OAZ knockdown, while anti-CCL2 antibodies completely counteracted the effect of OAZ silencing.

Pre-B-cell leukemia homeobox 1 (Pbx1)-d, an isoform of Pbx1, is a dominant negative mutation located in murine NZM2410 lupus susceptibility locus Sle1a1, with a deletion of DNA binding exon 6 and Hox binding exon 7 [40]. The amino acid sequence of mouse Pbx1 is identical to that of the human protein, which preserves self-renewal of hematopoietic stem cells and blocks lineage-specific differentiation [49]. Evidence shows that Sle1a1 MSCs express higher levels of Pbx1-d than normal MSCs, resulting in the generation of activated auto-reactive CD4^+^ T cells and increased anti-chromatin IgG production by B cells [40]. Pbx1-d mutation is found more frequently in SLE patients than in normal controls [50], implying that Pbx1-d also works in humans to regulate MSC function.

p16^INK4A^, an inhibitor of cyclin-dependent kinase (CDK)4 and CDK6, is closely associated with senescence of MSCs [51]. The expression of p16^INK4A^ has been shown to significantly increase in MSCs from SLE patients [52], which could account for the prominent senescence of lupus MSCs, as characterized by disordered cytoskeleton distribution and reduced immunoregulatory ability. Moreover, MSCs with p16^INK4A^ knockdown express elevated levels of TGF-β, leading to an increased percentage of Treg cells when cultured with purified CD4^+^ T cells. Therefore, p16^INK4A^ may cripple the function of SLE MSCs by both the induction of cell senescence and the inhibition of TGF-β secretion.

### Indirect evidence of genetic involvement

PD-1 has been identified as a lupus susceptibility gene in European and Mexicans since 2002 [53]. The risk allele A of the intronic single nucleotide polymorphism (rs1033438163) alters a binding site for the runt-related transcription factor 1 (RUNX1) and inhibits the expression of PD-1. Decreased expression of PD-1 may impair MSC suppression of p38, ERK, and Akt phosphorylation signaling transduction in B-cell receptor activated B cells, leading to the loss of peripheral self-tolerance in B cells [23].

Low affinity immunoglobulin gamma Fc region receptor II-b (FcγRIIB), a low affinity receptor usually expressed on B cells and myeloid DCs, is another lupus susceptibility gene in different populations [54]. Recently, this gene has been proven to be expressed on MSCs [55]. FcγRIIB-deficient MSCs are less potent at suppressing dendritic cell maturation and the antigen-specific T-cell response, along with reduced expression of immunosuppressive factors such as PGE2 and cyclooxygenase-2.

STAT protein family mediates IFN signaling responses and plays a role in maintaining immune tolerance. Both STAT1 and STAT4 have been reported to be related to autoantibody production and renal involvement in either lupus mice or SLE patients [56–58]. A risk allele of STAT4 has been found to be over-expressed in SLE mesenchymal cells [59]. Meanwhile, down-regulation of STAT1 abrogates MSCs’ immunosuppressive capacity via the inhibition of IDO production, while over-expression of STAT1 significantly enhances IDO production in MSCs [60]. Thus, STAT proteins may serve as potential regulators of lupus MSCs.

Several cytokines and chemokines, such as IL-6, IL-7, IDO, and CCL2, have been found to be down-regulated in MSCs from SLE patients [6, 17, 39]. The impaired ability of MSCs to secrete these regulatory factors may be attributed to the genetic background of SLE patients and accordingly weakened MSC function. For example, IL-6 reduction restrains MSC-induced macrophage polarization, while lack of IDO leads to insufficiency of the down-regulation of T-cell proliferation.

Factors that are highly expressed in serum from SLE patients may also play a role in the regulation of MSC function. Evidence shows that TNF-α could increase MSC migration and invasion via activation of the nuclear factor kappa-light-chain-enhancer of activated B cells (NF-κB) signaling pathway [61, 62]. Meanwhile, IFN-γ has been identified to stimulate IDO production in MSCs via the STAT1 signaling pathway [17], contributing to an enhanced immunosuppressive capacity of normal MSCs inhibiting the proliferation of lupus T cells. Recently, leptin and neutrophil-activating peptide 2 (NAP-2) have been shown to promote MSCs senescence through the activation of PI3K/Akt signaling pathway [63].

## Conclusions

SLE MSCs display impaired immunosuppressive capacity to not only acquired immunity but also innate immunity. A lot of genetic factors may contribute to the dysfunction of MSCs. Delineation of the genetic mechanism may not only be helpful to clarify disease pathogenesis but also beneficial for the enhancement of MSC efficacy. With continued research, genetic engineering to modify MSCs with improved immunosuppressive functions will be achieved in the near future.

## Abbreviations

AAM/M2: Alternatively activated macrophages
BM: Bone marrow
BMP: Bone morphogenetic protein
CCL2: Chemokine C-C motif ligand 2
CDK: Cyclin-dependent kinase
DC: Dendritic cell
FcγRIIB: Gamma Fc region receptor II-b
IDO: Indoleamine 2,3-dioxygenase
IFN: Interferon
IL: Interleukin
iNOS: Inducible nitric oxide synthase
MMP: Matrix metalloproteinase
MSC: Mesenchymal stem cell
NAP-2: Neutrophil-activating peptide 2
NF-κB: Nuclear factor kappa-light-chain-enhancer of activated B cell
NK: Natural killer
NO: Nitric oxide
OAZ: Olfactory 1/early B cell factor-associated zinc-finger protein
Pbx1: Pre-B cell leukemia homeobox 1
PD-1: Programmed death 1
PDL-1: Programmed death ligand-1
PGE2: Prostaglandin E2
RUNX1: Runt-related transcription factor 1
SLE: Systemic lupus erythematosus
STAT: Signal transducer and activator of transcription
Tfh: T follicular helper
TGF-β1: Transforming growth factor beta1
TNF-α: Tumor necrosis factor α
Treg: T regulatory
